# Ectopic Expression of *Hrf1* Enhances Bacterial Resistance via Regulation of Diterpene Phytoalexins, Silicon and Reactive Oxygen Species Burst in Rice

**DOI:** 10.1371/journal.pone.0043914

**Published:** 2012-09-06

**Authors:** Wenqi Li, Min Shao, Weigong Zhong, Jie Yang, Kazunori Okada, Hisakazu Yamane, Lei Zhang, Guang Wang, Dong Wang, Shanshan Xiao, Shanshan Chang, Guoliang Qian, Fengquan Liu

**Affiliations:** 1 College of Plant Protection, Nanjing Agricultural University, Nanjing, China; 2 Key Laboratory of Integrated Management of Crop Diseases and Pests (Nanjing Agricultural University), Ministry of Education, Nanjing, China; 3 Institute of Food Crops, Jiangsu Academy of Agricultural Sciences, Nanjing, China; 4 Biotechnology Research Center, The University of Tokyo, Tokyo, Japan; National Taiwan University, Taiwan

## Abstract

Harpin proteins as elicitor derived from plant gram negative bacteria such as *Xanthomonas oryzae* pv. *oryzae* (*Xoo*), *Erwinia amylovora* induce disease resistance in plants by activating multiple defense responses. However, it is unclear whether phytoalexin production and ROS burst are involved in the disease resistance conferred by the expression of the harpin_Xoo_ protein in rice. In this article, ectopic expression of *hrf1* in rice enhanced resistance to bacterial blight. Accompanying with the activation of genes related to the phytoalexin biosynthesis pathway in *hrf1*-transformed rice, phytoalexins quickly and consistently accumulated concurrent with the limitation of bacterial growth rate. Moreover, the *hrf1*-transformed rice showed an increased ability for ROS scavenging and decreased hydrogen peroxide (H_2_O_2_) concentration. Furthermore, the localization and relative quantification of silicon deposition in rice leaves was detected by scanning electron microscopy (SEM) and energy-dispersive X-ray spectrometer (EDS). Finally, the transcript levels of defense response genes increased in transformed rice. These results show a correlation between *Xoo* resistance and phytoalexin production, H_2_O_2_, silicon deposition and defense gene expression in *hrf1*-transformed rice. These data are significant because they provide evidence for a better understanding the role of defense responses in the incompatible interaction between bacterial disease and *hrf1*-transformed plants. These data also supply an opportunity for generating nonspecific resistance to pathogens.

## Introduction

Bacterial blight is one of the most destructive rice diseases. It is caused by *Xoo*, and results in 10% to 80% yield losses, endangering worldwide food security [1]. An economically effective and environmentally sound approach to control disease is the utilization of cultivars that possessed durable, nonspecific, broad-spectrum resistance by incorporating an elicitor protein harpin-encoding gene into susceptible rice with good agronomic traits [2]. However, we still know little about the signal transduction network of disease resistance induced by harpin proteins in plants.

As one kind of special compound, harpin protein elicitors provide a potential opportunity for the generation of durable broad-spectrum disease resistance in plants. Harpin, protein elicitors induce a hypersensitive response and multiple defense responses in non-hosts and determine pathogenicity in host plants [3], [4]. Harpin_Ea_, purified from the gram-negative plant pathogenic bacteria *Erwinia amylovora*, activates ROS burst, salicylic acid (SA) and jasmonic acid (JA)/ethylene (ET) signal transduction pathways, then confers different plants (such as tobacco, rice and *Arabidopsis thaliana*) systemic acquired resistance (SAR) and induced acquired resistance (ISR) both by exogenous application and ectopic expression in plants, resulting in nonspecific resistance to fungal and bacterial diseases [3]–[7]. Harpin_Xoo_ protein, derived from *Xoo* strain JXOIII possesses the basic elicitor characteristics of harpin protein that induce multiple defense responses in plants, such as systemic acquired resistance, hypersensitive response [2], [8]. The exogenous application of harpin_Xoo_ induced the activities of defense-related enzymes and increased resistance to Tobacco Mosaic Virus (TMV), *Sclerotinia sclerotiorum* (lib.) de Bary of rape and tomato *Botrytis cinerea* Pers. [9]. Moreover, expression of *hrf1* conferred rice highly resistance to major *Magnaporthe grisea* (*M. grisea*) races in rice growing areas by enhancing the expression levels of defense-related genes and increasing the silicon content [2]. Genetic transformation of the harpin_Xoo_-encoding gene in cotton improved resistance to *Verticillium dahliae* by triggering the generation of H_2_O_2_ and increasing the expression of defense-related genes [10]. ROS production in tobacco suspension cells elicited by exogenous application of harpin_Ea_, which are signal molecules mediate phytoalexin biosynthesis, induced the expression of defense-related genes and the hypersensitive response [11]. These defense responses induced by harpin proteins consisted of a complicated defense signal transduction network, which utilizes mutual coordination to enhance disease resistance in plants [2], [10], [12]. However, it is still not known whether ROS burst is involved in the disease resistance to bacterial pathogens conferred by harpin proteins in rice.

Phytoalexins, low molecular weight secondary metabolites are produced by host plants to respond to the infection of the fungal blast pathogen *M. grisea* and the bacterial leaf blight pathogen *Xoo*. Phytoalexins function as antimicrobials in destroying the growth and development of pathogens at infection sites [13], [14]. Rice produces 15 phytoalexins, including momilactones A (MA) and B (MB), phytocassanes A to E (PA to PE), oryzalexins A to F, and oryzalexin S, and flavonoid phytoalexin, sakuranetin [15]. These compounds quickly accumulate and exhibit antibiotic activity to inhibit the invasion of the rice-blast pathogens *M. grisea* and *Rhizoctonia solani* in incompatible rice [14], [16], [17]. Slight phytoalexin production is present in the healthy leaves of both monocotyledonous model plants rice and dicotyledonous model plants (e.g., *Arabidopsis thaliana*) under normal growth conditions, but there is an increase in production in both susceptible and resistant plants in response to attack by pathogens, such as bacterium *Pseudomonas syringae*, the necrotrophic fungi *Alternaria brassicicola* and *Botrytis cinerea* and the blast fungi *M. grisea* [15], [18]. In contrast, more highly and rapidly accumulated major phytoalexins, such as MA, MB, and PA to PE, contribute to the resistance to blast fungus in resistant rice, compared with the delayed induction of phytoalexin biosynthesis in susceptible rice plants [15], [19], [20]. So far, data regarding the accumulation and fate of phytoalexin biosynthesis in incompatible interactions between bacterial disease and rice is not well studied.

We have isolated and cloned *hrf1* from *Xoo*. Transgenic *hrf1* rice line NJH12 showed highly durable nonspecific resistance to all major *M. grisea* and rice false smuts, as well as enhanced drought tolerance by activating the expression levels of defense-related genes, and increased leaf silicon content and ROS-scavenging ability [2], [21]. On this basis, we focused on the rate of phytoalexin biosynthesis and H_2_O_2_ generation in *hrf1*-transformed rice with resistance to *Xoo*. In this study, these results suggested that *hrf1*-transformed plants showed an increased resistance to the main bacterial *Xoo* strains. Moreover, we first demonstrated that the ectopic expression of *hrf1* significantly enhanced the accumulation of phytoalexin production in rice after infection with *Xoo*, accompanying the induction of the transcript levels of genes involved in phytoalexin biosynthesis pathway. The change in the H_2_O_2_ concentration in *hrf1*-transformed rice was less when compared with that in the wild-type R109. Moreover, in accordance with the inhibition of H_2_O_2_ generation, activation of superoxide dismutase (SOD), peroxidase (POD) and catalase (CAT) significantly increased. Furthermore, the expression levels of defense-related genes were elevated in the transgenic *hrf1* rice, especially during interaction with *Xoo*.

## Materials and Methods

### Plant materials and pathogen inoculation

The T3 homozygous transgenic line NJH12 and the wild-type R109 were planted in a field after sprouting cultivation. R109 (*Oryzae sativa* subsp. *Japonica*) was susceptible to most *Xoo* strains, such as PXO79, PXO99 and JXOV. To evaluate the resistance to bacterial blight disease in rice, plants were inoculated with the Philippine *Xoo* strains PXO79 and PXO99 and Japanese strain JXOV at the booting stage by the leaf-clipping method [22]. Disease was scored using the 0 to 5 scale rating system by measuring the percentage lesion area (lesion length/leaf length) at 14 day post-inoculation (dpi). In this rating system, no obvious lesion in the leaves indicates at rating of 0 (high resistance), a lesion area less than 10% indicates a rating of 1 (resistance), a lesion area greater than or equal to 10% and less than 20% indicates a rating of 2 (modest resistance), a lesion area greater than or equal to 20% and less than 50% indicates a rating of 3 (modest susceptibility), a lesion area greater than or equal to 50% and less than 75% indicates a rating of 4 (susceptibility), and a lesion area greater than or equal to 75% indicates a rating of 5 (high susceptibility). *Xoo* growth rates in rice leaves were determined by counting colony-forming units [22].

### Extraction and phytoalexin quantification

For phytoalexin quantification in rice leaves inoculated with *Xoo*, leaves were detached at the booting stage after the indicated time period, and 0.1 g of each leaf cut from and frozen at −80°C until use. Leaf samples were mixed with 40 volumes of 70% methanol and heated for 5 min at boiling in a long glass tube with a screw cap. The extract was transferred to a new tube, and the residue was re-extracted twice with 20 volumes of 70% methanol. The combined extracts were concentrated to dryness. The residue was re-suspended in 0.5 ml of methanol and was subjected to HPLC-ESI-MS/MS for phytoalexin presence. HPLC-ESI-MS/MS was composed of an API-3000 with an electrospray ion source and an Agilent 1100 HPLC instrument equipped with a PEGASIL ODS column. Phytoalexin levels were determined with the combination of the precursor and productions (*m/z* 317/299 for PA, PD, and PE; *m/z* 335/317 for PB; *m/z* 315/271 for PC; *m/z* 315/271 for MA; *m/z* 331/269 for MB) in the MRM mode. The retention times of PA, PB, PC, PD, PE, MA and MB were 4.8, 4.2, 3.8, 5.9, 5.3, 6.4 and 4.9 min, respectively [15], [23].

### Measurement of H_2_O_2_ production

The production of H_2_O_2_ in fresh weight for both NJH12 and R109 was measured with a commercial H_2_O_2_ detection kit (Nanjing Jiancheng Bioengineering Institute, Nanjing, China) using the method described by Miao *et al* (2010). The samples were obtained from plants at different time periods 0, 0.5, 1, 1.5, 3 and 5 h after inoculation with *Xoo* PXO79 at the booting stage. Absorbance values were detected at 412 nm for the titanium-peroxide complex. The absorbance values were calibrated to a standard graph generated with known H_2_O_2_ content. The experiment was repeated three times.

### Measurement of malonyldialdehyde (MDA) content

MDA content was determined as described previously [24]. The samples were obtained from the plants at different time periods 0, 0.5, 1, 1.5, 3 and 5 h after inoculation with *Xoo* PXO79 at the booting stage. About 0.5 g of fresh leaves was homogenized in 5 ml of 10% (v/v) trichloroacetic acid, and the homogenate was centrifuged at 4,000 rpm for 10 min. Aliquots of the supernatants were boiled at 95°C for 25 min with 5 ml of 0.65% 2-thiobarbituric acid (TBA) and then measured at 532 nm.

### Measurements of antioxidant enzyme activities

Approximately 0.05–0.1 g of fresh leaves of NJH12 and R109 at different time periods 0, 0.5, 1, 1.5, 3 and 5 h after inoculation with *Xoo* PXO79, were homogenized in 0.45–0.9 ml of sterilized saline water at 0–4°C, and then 10% homogenate was obtained using a laboratory bead beater for 3 min. The sample was centrifuged for 10 min at 3000 rpm, and the resulting supernatant was transferred into tube by pipettor for detection of SOD, POD and CAT activity. The whole extraction procedure was carried out at 4°C. All reactions were replicated three times or more.

The activity of SOD was estimated by a method based on nitroblue tetrazolium (NBT) photoreduction modified from Jiang and Zhang (2001). The 3 ml reaction mixture was composed of 50 mM potassium phosphate (pH 7.8), 0.1 mM EDTA, 13 mM methionine, 75 µM NBT, 2 µM riboflavin, 0.1 mM EDTA and 100 µl supernatant. The mixtures were illuminated in glass test tubes for 10 min, and the absorbance of the mixtures at 560 nm was quickly determined with a spectrometer.

The activity of POD was measured at 530 nm in a reaction mixture containing 0.1 ml supernatant, 2 ml of 0.2 M acetate buffer (pH 4.8), 0.2 ml of 3% H_2_O_2_, and 0.2 ml of 0.04 M benzidine [21].

The activity of CAT was quantified by measuring the disappearance of H_2_O_2_ at 240 nm for 3 min. The reaction mixture contained 50 mM potassium phosphate buffer (pH 7.0), 10 mM H_2_O_2_ and 200 µl supernatant in a 3 ml volume. One unit of CAT activity was defined as causing the decomposition of 1 µmol H_2_O_2_ mg^−1^ protein min^−1^ at pH 7.0 [24].

### SEM and EDS analysis

For the SEM and EDS analysis, we mainly referred to the methods of Hayasaka, Fujii & Ishiguro [25]. The flag leaves at the ripening stage of rice plants were prepared. The middle segments of the leaf (1 cm×1 cm) were immediately dehydrated in a graded ethanol series (50, 70, 80, 90 and 100%). The specimens were mounted on aluminum stubs by carbon double-faced adhesive tape, coated with gold, and the morphological structure of silicified cells and papillae was examined by SEM at an accelerating voltage of 20 kV. The relative content of silicon was determined with an EDS combined with the SEM.

### Quantitative real-time polymerase chain reaction (qRT-PCR) analysis

The leaf fragments next to the bacterial infection sites at different time periods after inoculation were used for total RNA extraction at the booting stage. Total RNA obtained from the *hrf1*-transformed plant NJH12 and R109 using the Trizol reagent (TaKaRa, Dalian, China) following the manufacturer's protocol and then treated with RNase-free DNase (TaKaRa, Dalian, China). QRT-PCR was performed on the Applied Biosystems 7500 Real Time PCR System and SYBR Premix Ex Taq™ (TaKaRa, Dalian, China) according to the manufacturer's instructions. The rice gene *EF-1a* was used as the internal reference gene to standardize the RNA sample for evaluating relative expression levels. For qRT-PCR assays, three independent biological samples were used, accompanied by each repetition having three technical replicates with a gene-specific primer (Table S1).

## Results

### Ectopic expression of *hrf1* significantly increased resistance to *Xoo* strains in rice

**Figure 1:**
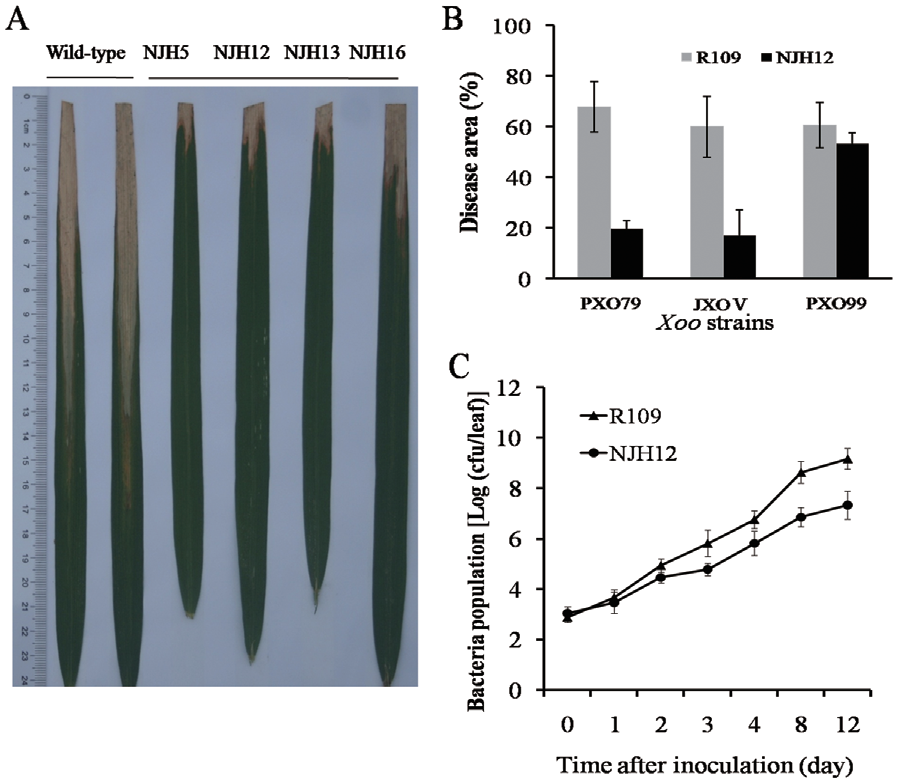
Increased resistance to *Xoo* strain PXO79 in the *hrf1*-transformed rice NJH12 at the booting stage. (A) Resistance phenotypes of NJH12 and wild-type R109 to PXO79. (B) Disease area of NJH12 and R109 in response to *Xoo* strains. (C) Growth of PXO79 in leaves of NJH12 and R109. Bacterial populations were determined by counting colony-forming units (CFU) at each time point. Data represent the means (three to five replicates) ± standard deviation.

We previously reported that the harpin_Xoo_-encoding gene *hrf1*, derived from *Xoo* and driven by the constitutive 35S promoter, was transferred into the *japonica* rice cultivar R109 by *Agrobacterium*-mediated transformation. The rice cultivar R109 is one of the dominant cultivated varieties with a high susceptibility to rice blast and bacterial blight in the Jiangsu province. The obtained homozygous T3–T7 transgenic NJH12 lines strongly enhanced durable nonspecific resistance to the main four *M. grisea* races by inhibiting appressorium formation in the Yangtze River region [2]. To examine whether *hrf1* confers rice resistance to bacterial disease, we chose the T3 transgenic line NJH12 to evaluate resistance to bacterial *Xoo* strains at the booting stage. Pathogen inoculation analysis demonstrated that the NJH12 line showed significantly enhanced resistance to the PXO79 strain, with the average disease area was 16.72%, compared with an average 67.78% in the wild-type R109 (Figure 1A, B). A bacterial growth analysis indicated that the growth rate of PXO79 in the resistant NJH12 line was 3.02- to 69.18-fold lower (*P*<0.05) than that in the wild type plants at 2 and 12 dpi (Figure 1C). These results showed that ectopic expression of *hrf1* enhanced resistance to *Xoo* strain PXO79.

Moreover, we examined the resistance spectrum to *Xoo* strains in the NJH12 line, which was inoculated with the PXO99 and JXO V strains respectively. The disease investigation results show that the NJH12 was more resistant to the JXO V strain, the disease areas were only 28.62% of those in the wild-type R109 (Figure 1B). Simultaneously, transgenic plants were more resistant to PXO99, compared with R109 plants (Figure 1B). Shao and associates have proved that *hrf1* transferred into NHJ12 plants by PCR and Southern blot, and harpin protein has been detected in NHJ12 leaves [2]. The data mentioned above strengthens the conclusion that *hrf1*, as an elicitor, induces broad-spectrum resistance to the main *Xoo* strains.

### Ectopic expression of *hrf1* in rice enhanced phytoalexin production

Phytoalexins are antimicrobials involved in fighting against bacterial and fungal disease invasion in plants. Previous reports have pointed out that diterpene phytoalexin levels, including MA, MB, and PA through PE (the main antimicrobials against fungal disease infection), were more rapidly and highly accumulated in resistant plants than that in susceptible plants [15], [20]. We deduced that ectopic expression of *hrf1* in rice activated the phytoalexin biosynthesis pathway, resulting in enhanced broad-spectrum disease resistance. The following two pieces of evidence lead to the hypothesis. First, the transcriptome profile in the leaves of the *hrf1*-transformed rice line was analyzed using a Biostar Rice-100S gene chip containing about 10,000 unigenes. The expression levels of some genes involved in secondary metabolite pathways significantly increased (unpublished). Among them, a cytochrome p450 gene showing a 114.6-fold increase of expression levels was identified. The *p450*-overexpressing rice lines showed increased phytoalexin accumulation, and resulted in broad-spectrum disease resistance (unpublished). Secondly, the leaf silicon content was dramatically enhanced in *hrf1*-transgenic plants whether at the tillering stage or the final harvest stage, inducing the accumulation of diterpenoids and flavonoid phytoalexins in rice [2], [26]. Therefore, we deduced that the ectopic expression of *hrf1* in rice likely activated the phytoalexin biosynthesis pathway by a set of molecular signals transduction.

**Figure 2:**
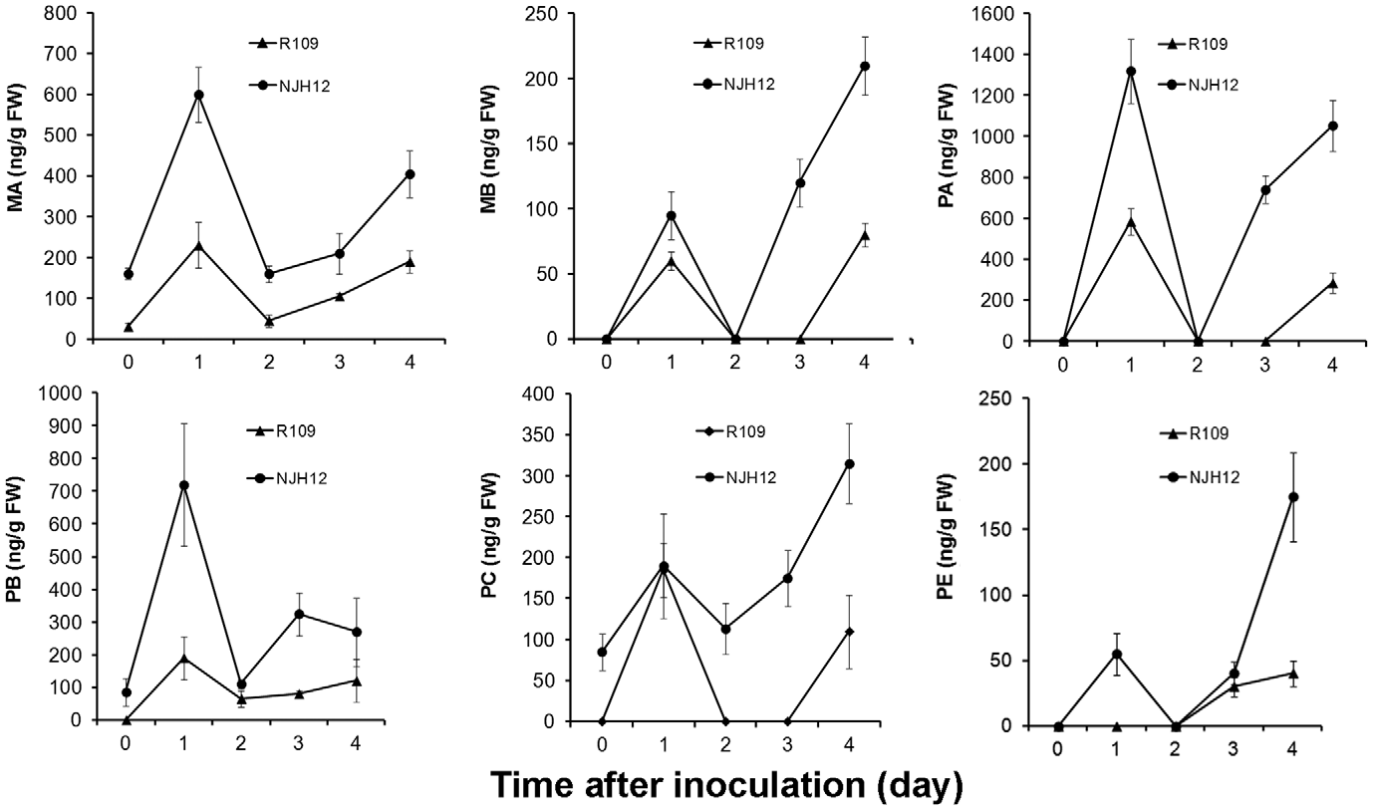
Accumulation of diterpenoid phytoalexins in NJH12 and R109 after inoculation with *Xoo* strain PXO79 at the booting stage. FW, fresh weight; Data represent the means (three replicates) ± standard deviation. MA, MB, PA, PB, PC and PE represent momilactone A, momilactone B, phytocassane A, phytocassane B, phytocassane C and phytocassane E, respectively. Phytocassane D was induced with a similar induction mode, but all values were lower than the other phytocassanes shown (data not shown).

Not only to test this hypothesis, but also to analyze the accumulation and rate of diterpene phytoalexin biosynthesis during the interaction between resistant rice and *Xoo*, we detected the accumulation of seven main diterpene phytoalexin components in NJH12 by HPLC-ESI-MS/MS using individual authentic chemicals as standards under normal growth and *Xoo* inoculation conditions. As Figure 2 shows, the levels of MA, PB and PC were higher in NHJ12, compared with those in R109 without inoculation. The data may demonstrate that *hrf1* acts as a positive regulator of the phytoalexin biosynthesis pathway. Then we analyzed the rate of phytoalexin biosynthesis in NJH12 and R109 after treatment with *Xoo* strain PXO79 at the booting stage. There was a distinct difference in the phytoalexin biosynthesis pattern between in NJH12 and R109, although both were obviously increased after *Xoo* infection. At 1 dpi, MA, MB, and PA through PE were accumulated to high levels in both plant lines. At 2 dpi, the levels were reduced to nearly 0 µg/g of fresh weight, and then again were elevated to maximal (Figure 2). However, all the phytoalexin accumulation in NJH12 was higher than that in R109 at 1 dpi, 3 dpi and 4 dpi, although at 2 dpi the levels tended to consistent with those in R109 (Figure 2). These results proved that *hrf1* expression remarkably enhanced the accumulation of phytoalexin production in the transgenic line NJH12 with inoculation or without. Obviously, phytoalexin productions were quickly accumulated to a high level at 1 dpi, and phytoalexins were continuously produced in the NJH12 plants after 2 dpi (Figure 2). This quick and sustainable response mechanism in phytoalexin biosynthesis may partly lead to enhanced disease resistance, in accordance with the accumulation pattern of phytoalexin production in the resistant line IL7 [15].

### The multiplication of *Xoo* was inhibited by exogenous application of phytoalexin production

**Figure 3:**
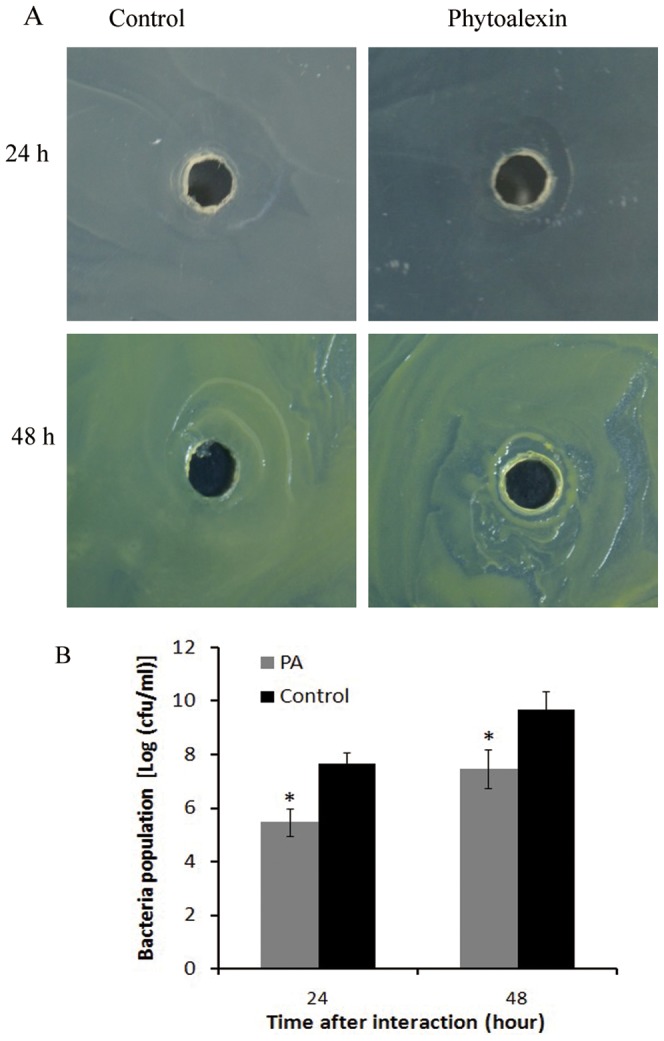
Phytoalexin production showed the antimicrobial activity to *Xoo*. (A) Inhibitation of phytoalexin against multiplication of *Xoo* PXO79. (B) Growth of *Xoo* PXO79 in liquid nutrient medium containing 200 ng/ml phytoalexins. Data represent the means (three replicates) ± standard deviation.

The effect of phytoalexin production on the multiplication of *Xoo* PXO79 in vitro was analyzed using agar diffusion test. Spread the 100 µl PXO79 solution at logarithmic growth phase on nutrient agar (NA) medium, make a hole at center of plate after drying, subsequently drop 30 µl 200 ng/ml phytoalexin solution into the hole. At 1 day after interaction, about 0.8 cm diameter inhibition zone developed in plate treatment with phytoalexin production, at 2 day phytoalexin production significantly inhibited multiplication of PXO79 resulted in a clear inhibition zone (Figure 3A). Moreover, the diameter of inhibition zone was unchanged at 3 or 4 day. Simultaneously, there was no inhibition zone in plate without phytoalexin production. Further, multiplication of *Xoo* in liquid nutrient medium containing 200 ng/ml phytoalexin was inhibited (Figure 3B). These results partly suggested that phytoalexin production showed antibiotic activity to *Xoo* PXO79.

### The transcripts of genes related to phytoalexin biosynthesis were obviously elevated in NJH12

**Figure 4:**
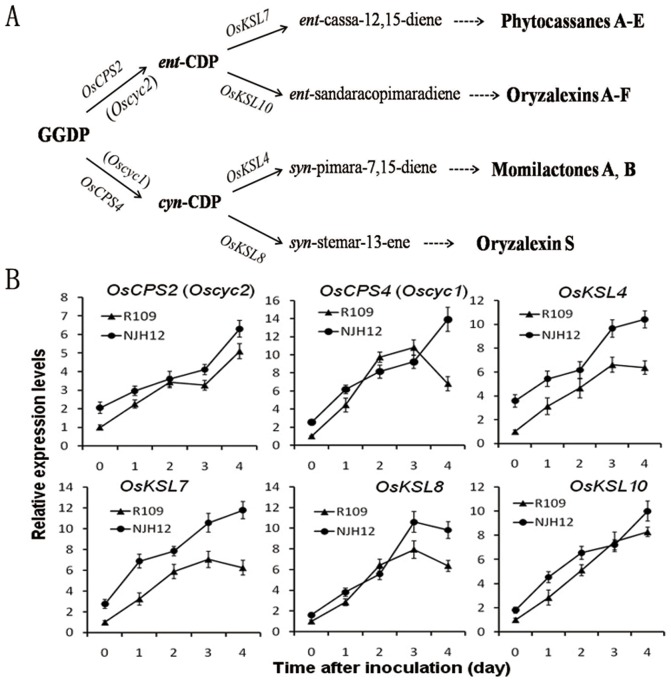
Induced expression of genes involved in the biosynthesis of diterpene phytoalexins in NJH12 and R109 after inoculation with *Xoo* strain PXO79 at the booting stage. (A) Proposed biosynthetic pathways for rice diterpene phytoalexins [15], [28]. GGPP: (E,E,E)-geranylgeranyl diphosphate, *ent*-CDP: *ent*-copalyldiphosphate, *syn*-CDP: *syn*-copalyl diphosphate. (B) Transcript levels of genes for rice diterpene phytoalexin biosynthesis in NJH12 and R109 after inoculation with *Xoo* strain PXO79 were determined by qRT-PCR. Bars represent the means ± SD (three replicates).

To examine whether the ectopic expression of *hrf1* activated the pathway of phytoalexin biosynthesis, resulting in more phytoalexin production in responding to disease infection, the expression levels of six genes related to phytoalexin biosynthesis pathway were quantified by qRT-PCR in the transgenic line NJH12 after inoculation with the *Xoo* strain PXO79. A proposed biosynthetic pathway for rice diterpene phytoalexins has been accepted (Figure 4A). In this pathway, there are the four classes of rice diterpene cyclase genes, including *OsCPS2*, *OsCPS4*, *OsKSL4*, *OsKSL7*, *OsKSL8* and *OsKSL10* six genes, which function in momilactones, phytocassanes and oryzalexin biosynthesis [15], [27], [28]. These quantitative results showed that the expression levels of *OsCPS2*, *OsCPS4*, *OsKSL4* and *OsKSL7* in NJH12 without inoculation were sharply induced (Figure 4B). Under the inoculation conditions, the expression level of *OsCPS2* in NJH12 was higher than that in R109 after 2 dpi (although both transcript patterns were consistent), and elevated about 4-fold at 4 dpi compared with that 0 dpi (Figure 4B). However, the expression patterns of *OsKSL4* and *OsKSL7*, which functioned in phytocassanes biosynthesis and momilactones biosynthesis, respectively, were almost identical. Both transcripts transiently increased at 1 dpi and decreased at 2 dpi, with a subsequently sharp elevation, reaching to a maximum at 4 dpi in NJH12. In contrast, transiently increased transcripts of *OsKSL4* and *OsKSL7* in R109 were not observed at 1 dpi, but obviously decreased at 4 dpi (Figure 4B). Interestingly, the expression levels of *OsCPS4*, *OsKSL4*, *OsKSL7*, *OsKSL8* and *OsKSL10* in R109 at 2 dpi were remarkably higher than those in the transgenic line NHJ12, and the transcripts of the five genes in NJH12 were also much higher and increased more sharply than that in R109 at 4 dpi (Figure 4B). In conclusion, the expression levels of all six genes in NJH12 before 1 dpi were higher than those in R109, lower at 2 dpi, and elevated to maximum at 4 dpi, higher than that those in R109 (Figure 4B). We also analyzed the expression level of these six genes in NJH5, showed similar result with that in NJH12 (Figure S1). These results proved that ectopic expression of *hrf1* in rice activated the expression of genes-related to the phytoalexin biosynthesis pathway. A consistent response pattern has been demonstrated in resistant rice: the expression levels of the six genes were transiently elevated with simultaneously activated phytoalexin biosynthesis [15].

### Inhibition of ROS generation in NJH12

**Figure 5:**
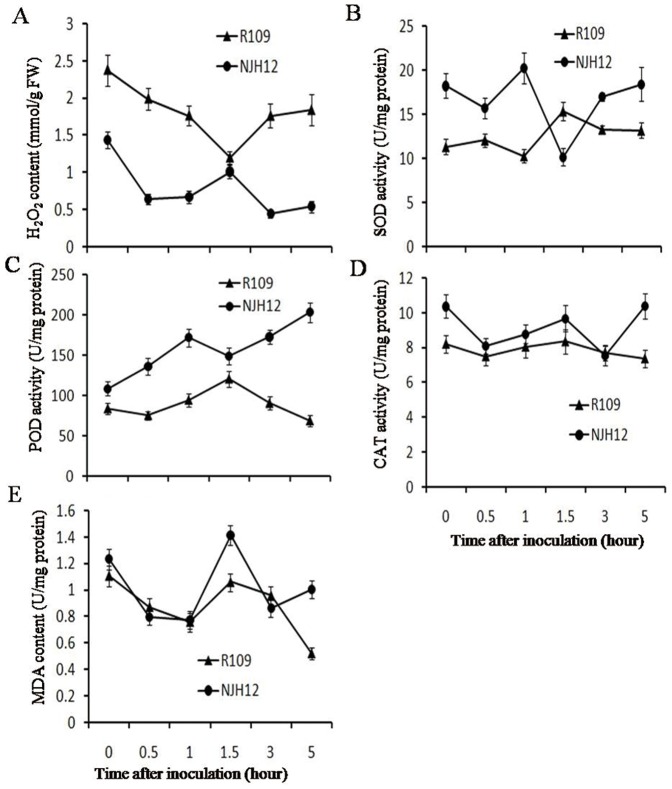
Generation of ROS in leaves of NJH12 and R109. (A) H_2_O_2_ content (mmol/g FW) in leaves of NJH12 and R109. FW: fresh weight. (B) ROS-scavenging ability in NJH12 and R109 after inoculation with *Xoo* strain PXO79. Bars represent the means ± SD (three replicates).

H_2_O_2_ is a main ROS that mediates the phytoalexin biosynthesis and induced by harpin protein in plants [11]. To test whether the expression of *hrf1* in rice induced the H_2_O_2_ burst, we measured H_2_O_2_ accumulation in both NHJ12 and R109 leaves under normal growth and inoculation conditions. The content of H_2_O_2_ in NJH12 was 1.44 mmol/g fresh weight lower than the 2.38 mmol/g fresh weight in R109 under normal growth conditions (Figure 5A). After inoculation with *Xoo*, the rate of H_2_O_2_ generation in NJH12 at different time periods was still significantly reduced compared with that in R109, although the levels of both were decreased (Figure 5A). The antioxidant enzymes SOD, POD and CAT are important parts of the ROS-scavenging mechanisms in plants [29]. MDA is an important intermediate in ROS scavenging, and a high level of MDA induces PCD and is toxic to plant cells [29], [30]. Accordingly, the activation of SOD and POD in NJH12 were obviously increased at different time periods compared with that in R109, although the activation of SOD in NJH12 was lower than that in R109 at 1.5 h (Figure 5B, C). The activation of CAT in NJH12 at most time periods also was higher than that in R109, while tended to equal at 3 h (Figure 5D). Moreover, the change of MDA content in NJH12 was consistent with that in R109 at most time periods, while the content was higher than that in R109 at 1.5 h and 5 h (Figure 5B). The results presented here show that the ectopic expression of *hrf1* in rice inhibited H_2_O_2_ accumulation by enhancing ROS-scavenging ability, which was consistent with the data by Peng *et al*. (2004) and Zhang *et al* (2011).

### Ectopic expression of *hrf1* changed localization and relative content of silicon deposition in rice leaves

**Figure 6:**
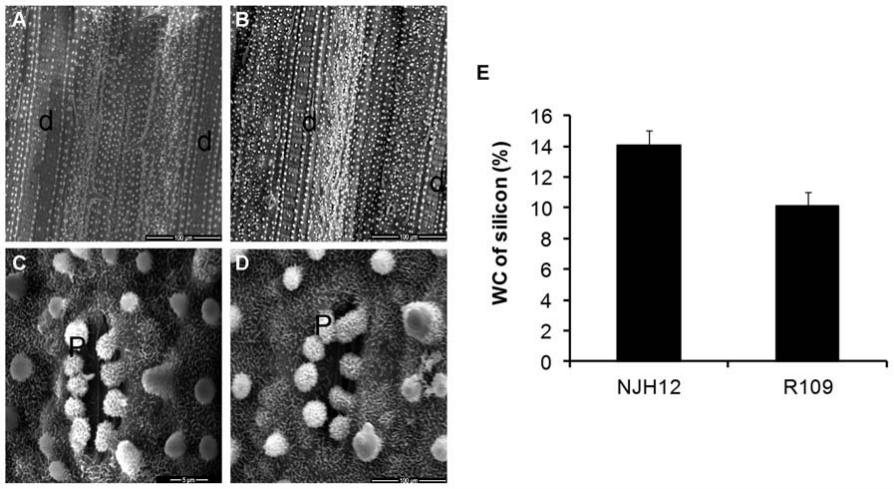
Localization and quantification of silicon deposition in the leaf of NJH12 and R109. Leaf epidermis of NJH12 ([A] and [C]) and R109 ([B] and [D]) at the ripening stage were observed by scanning electron microscopy. d: silicified dumbbell-shaped cell; p: silicified papillae; s: stoma. (A) and (C) are displayed on the same scale, and (B) and (D) are displayed on the same scale. Scale bar is included in the figures. (E) The weight concentration of silicon determined with an EDS at the leaf surface of NJH12 and R109. WC: weight concentration. Bars represent the mean ± SD (three replicates).

Silicified dumbbell-shaped cells and papillae are the two main types of silicon in rice leaf blades, which play an important role for resistance to pathogens [31]. Our previous report demonstrated that expression of *hrf1* in rice increased leaf silicon content at the tillering stage or the final harvest stage [2]. We detected the distribution of silicon deposition in flag leaves of both NJH12 and R109 at ripening stage by SEM. SEM analysis showed a clear morphological difference in the silicon deposition between NJH12 and R109. The most obvious difference was that there were two contiguous line silicified cells in NJH12, and there was one in R109 (Figure 6A, B). The accompanying gap between two silicified cells in NJH2 was longer than that in R109, and the numbers of papillae between two silicified cells in NJH12 was usually one, compared with two in R109 (Figure 6A, B). Moreover, the numbers of papillae reached to 34 in NJH12, which was more than 21 in R109 (at 30 µm×30 µm). The numbers of papillae around the stoma was usually 9 in NH12 and 7 or 8 in R109 (Figure 6C, D). The weight concentration of silicon detected by EDS on the leaf surface of NJH12 increased, compared with R109 (Figure 6E). The data showed here at least partly proved that the expression of *hrf1* in rice controlled distribution of silicon deposition.

### Ectopic expression of *hrf1* activated transcripts of the genes related to defense response

To test whether the enhanced broad-spectrum disease resistance in NJH12 accompanied the activation of SA- and JA-dependent defense pathways, we analyzed the expression levels of four known key genes related to these both pathways. The *NH1* (*Arabidopsis* homolog non-expressor of pathogenesis-related genes 1) gene functioned as a defensive signal transduction not only in SAR mediated by SA but also in ISR mediated by JA. Acidic pathogenesis-related (PR) protein 1 (*PR1a*; AJ278436) is involved in the SA signal pathway; lipoxygenase (*LOX*; D14000) and allene oxide synthase 2 (*AOS2*; AY062258) are involved in JA synthesis [22]. The expression of harpin in transgenic plants activated SAR and ISR mediated by SA [4], [5], JA or ET [6], respectively. However, it is unknown whether these defense responses are involved in the resistance to *Xoo* in NJH12.

**Figure 7:**
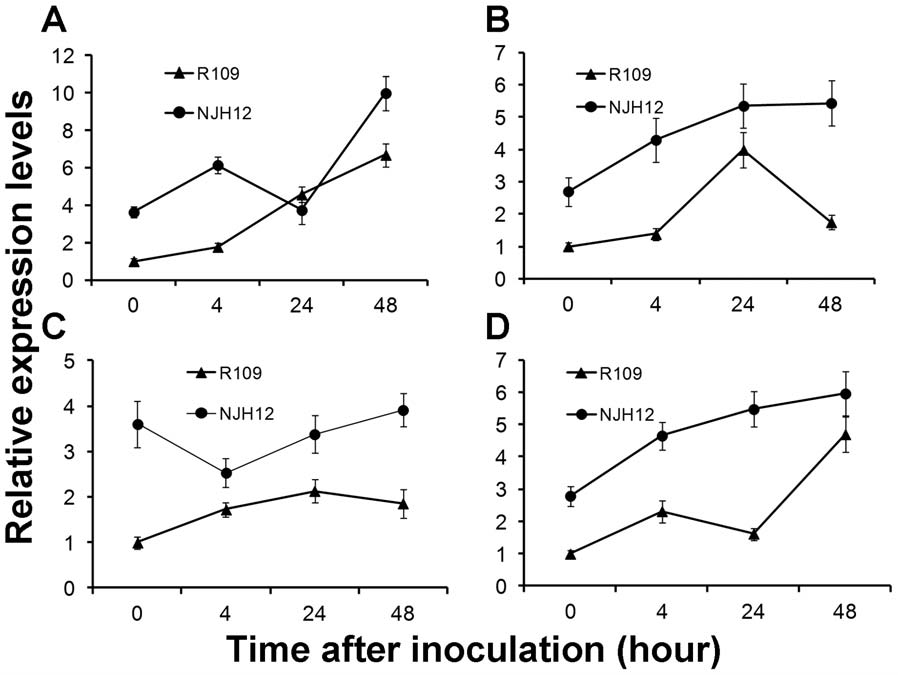
Expression analysis of four defense response-related genes in NJH12 and R109 after inoculation with *Xoo* strain PXO79 at the booting stage. (A) *AOS.* (B) *LOX*. (C) *NH1*. (D) *PR1-a*. Bars represent the mean ± SD (three replicates).

Relative expression analysis by qRT-PCR suggested the transcripts of four genes acted in two classes of defense signaling pathways significantly increased in NJH12 without inoculation, compared with those in R109, reached 3.622- (*AOS2*), 2.703- (*LOX*), 3.604- (*NH1*) and 2.794- (*PR1a*) fold (Figure 7), respectively. The gene expression analysis proved that ectopic expression of *hrf1* in rice remarkably induced the expression of genes related to the SA and JA signaling pathways, which was consistent with the results obtained in our previous study [2]. Moreover, accompanying *Xoo* infection, the transcripts of the four genes were 1.25 to 3.12-fold higher in the NJH12 line than in the R109 at most time points (Figure 7). These results further confirmed that the *hrf1* may have functioned as a positive regulator and induce host defense responses mediated by SA and JA, agreeing with data produced in transgenic harpin-encoding gene plants [2], [4], [10]. These data presented in this article suggested that *hrf1* may activate SAR and ISR mediated by the SA and JA signaling pathways, respectively during the course of resistance to bacteria *Xoo* infection.

## Discussion

During the past two decades, the biological function of harpin protein has been widely studied in both the monocotyledonous and dicotyledonous plants and is involved in enhancing growth, development and drought tolerance as well as increasing disease resistance [2], [10], [12], [21], [32]–[34]. In this article, we showed that ectopic expression of *hrf1* in rice significantly enhanced resistance to *Xoo* strains, accompanied by high and rapid induction of phytoalexins production and changed the distribution of silicified dumbbell-shaped cells and papillae. Moreover, ectopic expression of *hrf1* protected rice from oxidative damage resulting from inhibition of H_2_O_2_ generation via increasing the ability of ROS scavenging. These results presented in this study supported our conclusion that expression of *hrf1* conferred broad-spectrum disease resistance in rice by inducing multiple defense responses, such as the accumulation of phytoalexins, silicon and the activation of defense genes, and avoiding oxidative damage by the inhibition of H_2_O_2_ generation resulted from an increased ability for ROS scavenging.

Phytoalexins, as antimicrobial compounds, play an important in the biochemical defense response of plants to repress the multiplication of various fungi and bacteria *in vitro* as well as *in vivo* [15], [16], [35]. In contrast, less accumulation has been found in camalexin-deficient mutants, resulting in more susceptibility to fungus and bacterial disease compared with wild-type plants, such as *cyp79B2/cyp79B3* double mutants, *pad3-1* and *cyp71A13* mutants were susceptible to *A. brassicicola*, camalexin-deficient *pad4* and *pad2* had enhanced susceptibility to *P. syringae* [18], [36]. However, the role of phytoalexin production in resistance to bacterial diseases in rice is still unclear. In this article, we showed the induction of phytoalexin production during incompatible interaction between *hrf1*-transformed rice and *Xoo* accompanying the inhibition of bacteria growth rate (Figure 1, 2 and 3). As mentioned above, more transcripts of genes related to the phytoalexin biosynthesis have been quantified in the *hrf1* transgenic rice NHJ12 not only under normal growth conditions but also under *Xoo* strain invasion (Figure 4). There was insufficient phytoalexin production to inhibit *Xoo* multiplication at 1 dpi in wild-type R109. Accompanied by massive multiplication of bacteria, after 2 dpi, the expression levels of genes related to phytoalexin biosynthesis could not quickly synthesize sufficient phytoalexins to resist the multiplication of bacteria (Figure 1C, 2 and 4). In contrast, *Xoo* multiplication was suppressed by enough phytoalexins in *hrf1*-transformed plants at 1 dpi; at 2 dpi, excess phytoalexins inhibited bacterial growth, and at 3–4 dpi, phytoalexin production was continuously increased (Figure 1C, 2 and 4). The quick and sustainable induction of phytoalexins accumulation may play a critical role in bacterial disease resistance of *hrf1*-transformed plants. The sufficient phytoalexin production restricted fungus growth at 1–2 dpi in resistant rice and phytoalexins accumulated continuously at 3 and 4 dpi [15].

Interestingly, at 2 dpi, the expression levels of six genes related to phytoalexin biosynthesis in wild-type R109 plants were higher than those in *hrf1*-transformed rice, although the accumulation of phytoalexins is similar in both (Figure 2, 4). A possible interpretation is that phytoalexin production was rapidly synthesized in R109 at 2 dpi, and then detoxified by the large number of bacteria. For better survival in the host plant, the plant pathogens evolved the capability of phytoalexin detoxification for protection against impairment [37]. Many fungi have the ability to detoxify the phytoalexins in plants [38], [39], although the virulent *Arabidopsis thaliana* pathogen *Pseudomonas syringae* pv. *maculicola* strain ES4326 (*Psm* ES4326) has no capacity to degrade camalexin [40]. As mentioned above, phytoalexin detoxification may exist in the interaction of rice with *Xoo*.

It was well known that H_2_O_2_, as the main ROS functioned in defense responses as a signal transduction molecule [41]. In this article, accompanying the increase of ROS-scavenging ability (Figure 5B), H_2_O_2_ generation in the *hrf1*-transformed plant NJH12 both under normal growth and inoculation conditions was inhibited compared with that in wild-type R109, and vice versa (Figure 5A). Increased ROS-scavenging ability in *hrf1*-tranformed plants or with exogenous application of harpin_Xoo_ was obtained. Recently, enhanced ROS-scavenging ability in NJH12 has been shown under normal growth or drought stress conditions [21]. Expression of harpin_Xoo_ in transgenic tobacco showed increased resistance to fungal, bacterial and viral pathogens without the generation of hypersensitive cell death and reactive oxygen intermediate burst [42]. Moreover, the exogenous application of harpin_Xoo_ enhanced POD activation [9], which plays an important role in ROS-scavenging system. These results suggested that harpin_Xoo_ inhibited H_2_O_2_ generation by increasing ROS-scavenging ability. Therefore, we deduced that ROS burst may not functioned as an early defense event in the interaction between *hrf1*-transformed rice and *Xoo*. However, H_2_O_2_ production was stimulated by not only the exogenous applications of harpin_Ea_ and harpin_Pss_ protein in tobacco, *Arabidopsis* and sweet pepper, but also in transgenic cottons [10], [11], [43]. Therefore, harpin proteins regulation of the ROS signal may be dependent on different receptors [42]. For better understanding of the defense mechanisms activated by harpins, more progress is needed and underway.

Silicon takes part in plant growth and development as well as defense responses. The application of silicon increased rice disease resistance to blast and sheath blight by inducing of phytoalexin production [26]. At the same time, silicon-treated rice leaves significantly enhanced the activation of the protective enzymes POD and CAT and the content of MDA, resulting in reduced accumulation of H_2_O_2_ [44], [45]. Our results show that expression of *hrf1* in rice increased silicon relative content by changing the distribution of silicon deposition (Figure 6). We suggested that there may be a mutual relationship between silicon, phytoalexins and H_2_O_2_. Likely, the ectopic expression of *hrf1* in rice enhanced silicon accumulation, and subsequently, the silicon inhibited H_2_O_2_ generation and induced phytoalexin production.

In conclusion, our data support the idea that the ectopic expression of *hrf1* in rice regulated multiple defense responses, such as phytoalexins, silicon, H_2_O_2_ and defense-related genes, which likely cooperated in the induction of disease resistance. The data in this article provide evidence for better exploring the role of multiple defense responses induced by the harpin_Xoo_ in incompatible interaction of rice with bacterial disease, as well as supply a potential approach for generating the durable broad-spectrum disease resistance by utilization of harpin-encoding gene transgenic plants.

## Supporting Information

Table S1 **Primers used in this study for qRT-PCR analysis.**
(DOC)

Figure S1 **Induced expression of genes involved in the biosynthesis of diterpene phytoalexins in NJH5 and R109 after inoculation with** ***Xoo*** **strain PXO79 at the booting stage.** Transcript levels of genes for rice diterpene phytoalexin biosynthesis in NJH5 and R109 after inoculation with *Xoo* strain PXO79 were determined by qRT-PCR. Bars represent the means ± SD (three replicates).
(DOC)
