# Supplementary material for: Ectopic Expression of Hrf1 Enhances Bacterial Resistance via Regulation of Diterpene Phytoalexins, Silicon and Reactive Oxygen Species Burst in Rice
Source: PLoS One. 2012 Sep 6;7(9):e43914. doi: 10.1371/journal.pone.0043914 (PMC3435380; doi:10.1371/journal.pone.0043914)
Supplement: Table S1 — Primers used in this study for qRT-PCR analysis. (DOC) [file pone.0043914.s001.doc]

| Primers | Sequence (5’to 3’) | Production size (bp) | Accession Numbers |
| --- | --- | --- | --- |
| *EF1a*-F | ACAAGCTTGGAGGTATCGAC | 115 | Os03g0177400 |
| *EF1a*-R | CGCTCGGCCTTGAGCTTGTC |
| *OsCPS2*-F | CATGACAGAGAGGCTCATCA | 105 | AY602991 |
| *OsCPS2*-R | TGAGCTCATCAAGTGCGT |
| *OsCPS4*-F | CGGAACGTCTTGGATGGGCTC | 106 | AY530101 |
| *OsCPS4*-R | GCTCTTCAAGATTGCTGGTCG |
| *OsKSL4*-F | CGAGACATCCGCTTTGTAACT | 102 | AY616862 |
| *OsKSL4*-R | AGGCTTGTATATCATTGTATT |
| *OsKSL7*-F | GGTGTATGAAGGTTGAGGCA | 109 | DQ823354 |
| *OsKSL7*-R | GACATCACCGTACAGCCCTG |
| *OsKSL8*-F | GCAGGGAGGTCTTCTGGCACA | 131 | AB118056 |
| *OsKSL8*-R | TTACTCTTGCAGGTGCAGTGG |
| *OsKSL10*-F | GCATGCACTTATGATGTCTTA | 103 | DQ823355 |
| *OsKSL10*-R | CATATGGTTGTAGCAAAAGCC |
| *PR1*-F | AGGTATCCAAGCTGGCCAT | 92 | NM_001049624 |
| *PR1*-R | GAGCCTCACGTAGTCCTGC |  |  |
| *NH1*-F | CACGCCTAAGCCTCGGATTA | 121 | AY923983 |
| *NH1*-R | TCAGTGAGCAGCATCCTGACTAG |  |  |
| *AOS2*-F | CAATACGTGTACTGGTCGAATGG | 135 | NM_001055971 |
| *AOS2*-R | AAGGTGTCGTACCGGAGGAA |  |  |
| *LOX*-F | GCATCCCCAACAGCACATC | 114 | NM_001068734 |
| *LOX*-R | AATAAAGATTTGGGAGTGACATATTGG |  |  |

Table S1. Primers used in this study for qRT-PCR analysis.
