# Supplementary material for: Ectopic Expression of Hrf1 Enhances Bacterial Resistance via Regulation of Diterpene Phytoalexins, Silicon and Reactive Oxygen Species Burst in Rice
Source: PLoS One. 2012 Sep 6;7(9):e43914. doi: 10.1371/journal.pone.0043914 (PMC3435380; doi:10.1371/journal.pone.0043914)
Supplement: Figure S1 — Induced expression of genes involved in the biosynthesis of diterpene phytoalexins in NJH5 and R109 after inoculation with Xoo strain PXO79 at the booting stage. Transcript levels of genes for rice diterpene phytoalexin biosynthesis in NJH5 and R109 after inoculation with Xoo strain PXO79 were determined by qRT-PCR. Bars represent the means ± SD (three replicates). (DOC) [file pone.0043914.s002.doc]

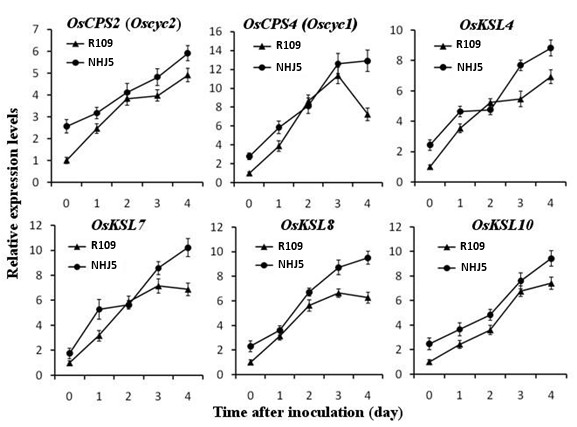


**Figure S1. Induced expression of genes involved in the biosynthesis of diterpene phytoalexins in NJH5 and R109 after inoculation with *Xoo* strain PXO79 at the booting stage.** Transcript levels of genes for rice diterpene phytoalexin biosynthesis in NJH5 and R109 after inoculation with *Xoo* strain PXO79 were determined by qRT-PCR. Bars represent the means ± SD (three replicates).
